# Tick vaccines and the control of tick-borne pathogens

**DOI:** 10.3389/fcimb.2013.00030

**Published:** 2013-07-09

**Authors:** Octavio Merino, Pilar Alberdi, José M. Pérez de la Lastra, José de la Fuente

**Affiliations:** ^1^SaBio, Instituto de Investigación en Recursos Cinegéticos IREC-CSIC-UCLM-JCCMCiudad Real, Spain; ^2^Department of Veterinary Pathobiology, Center for Veterinary Health Sciences, Oklahoma State UniversityStillwater, OK, USA

**Keywords:** tick-borne pathogens, vaccine, transmission-blocking, tick, vector

## Abstract

Ticks are obligate hematophagous ectoparasites that transmit a wide variety of pathogens to humans and animals. The incidence of tick-borne diseases has increased worldwide in both humans and domestic animals over the past years resulting in greater interest in the study of tick-host-pathogen interactions. Advances in vector and pathogen genomics and proteomics have moved forward our knowledge of the vector-pathogen interactions that take place during the colonization and transmission of arthropod-borne microbes. Tick-borne pathogens adapt from the vector to the mammalian host by differential gene expression thus modulating host processes. In recent years, studies have shown that targeting tick proteins by vaccination can not only reduce tick feeding and reproduction, but also the infection and transmission of pathogens from the tick to the vertebrate host. In this article, we review the tick-protective antigens that have been identified for the formulation of tick vaccines and the effect of these vaccines on the control of tick-borne pathogens.

## Introduction

Ticks are of great medical and veterinary importance as they can transmit a wide variety of infectious agents (de la Fuente et al., 2008a). The family Ixodidae comprises hard ticks of the *Amblyomma*, *Dermacentor*, *Rhipicephalus*, and *Ixodes spp*. that not only inflict direct damage to their host but also rank second to mosquitoes as vectors of disease. The *Ixodes ricinus* species alone transmits viruses, bacteria, and protozoa that cause in humans tick-borne encephalitis, Lyme disease, and babesiosis, respectively (de la Fuente et al., 2008a). In cattle, anaplasmosis caused by *Anaplasma spp*., and babesiosis, caused by *Babesia spp*., are two of the most important diseases transmitted by *Rhipicephalus spp*. ticks (Merino et al., 2011a).

Vector-borne diseases are on the increase and new infectious agents are also emerging leading to significant public health concerns as potential zoonotic disease threats (Parola and Raoult, 2001; de la Fuente and Estrada-Peña, 2012). Amongst other factors, climate change itself can have an adverse effect on the distribution of ticks and tick-borne diseases. It is predicted that more than 50% of tick species of the genus *Rhipicephalus* (*Boophilus*) could expand its range in Africa, with more than 70% of this range expansion linked to economically important tick species such as *R. appendiculatus, R. microplus*, or *R. decoloratus* (Estrada-Peña et al., 2006; Olwoch et al., 2007).

The ultimate goal of arthropod vector vaccines is the control of vector infestations and vector-borne diseases (VBD). The effect of vector vaccines on VBD could be obtained by (a) reducing vector populations and thus the exposure of susceptible hosts to vector-borne pathogens, (b) reducing the arthropod vector capacity for pathogen transmission, and, preferably, (c) a combination of these factors.

**Figure 1:**
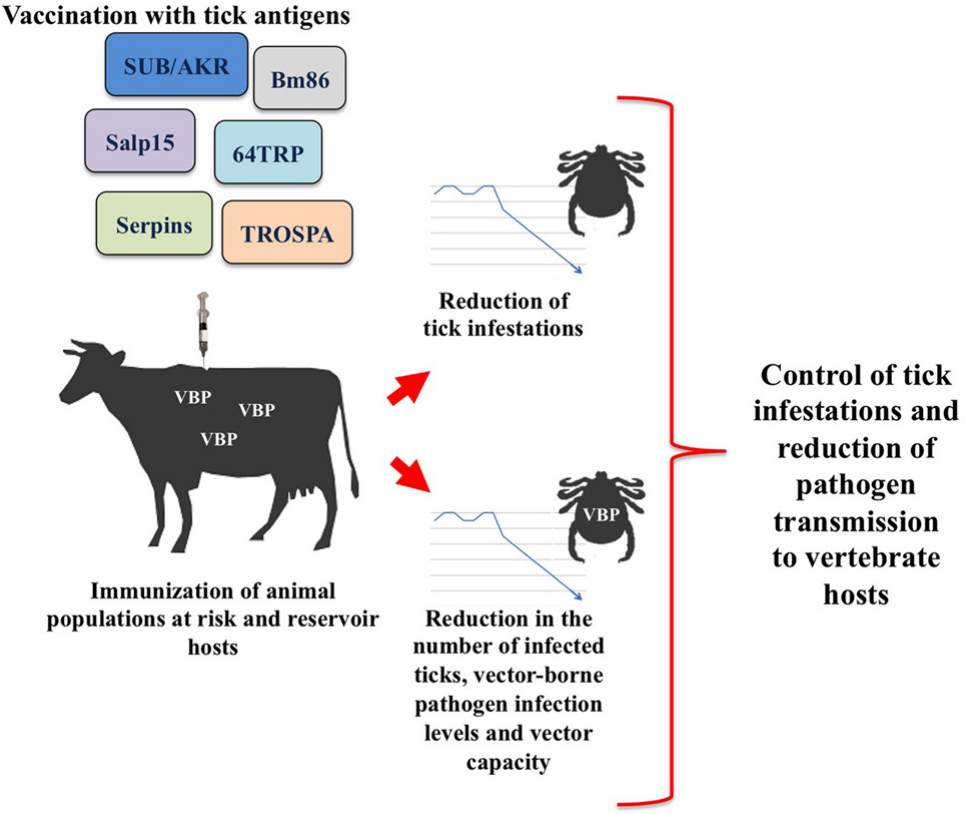
**Transmission blocking vaccines based on tick recombinant proteins aim to reduce vector infestations and the infection and transmission of vector-borne pathogens (VBP)**.

Herein, we review recent advances in tick vaccine development focused on discovery and characterization of tick protective antigens that impact on pathogen infection and transmission. Identification of molecules essential for both tick survival and pathogen infection and transmission will likely contribute to the discovery of novel vaccine strategies for the simultaneous control of ticks and tick-borne pathogens (Figure 1).

## Control methods for ticks and tick-borne diseases

A major component of integrated tick control has been the application of acaricides. However, their use has had limited efficacy in reducing tick infestations and is often accompanied by serious drawbacks, including the selection of acaricide-resistant ticks, environmental contamination and contamination of milk and meat products with drug residues (Graf et al., 2004; Ghosh et al., 2007).

An alternative host-targeted method involves the elimination of ticks from the host using baits impregnated with different compounds such as acaricides or antibiotics. Immature *I. scapularis* ticks were eliminated from mice using bait boxes impregnated with fipronil, therefore reducing the subsequent populations of nymphs and adults and thus reducing the proportion of ticks infected with the Lyme disease agent, *Borrelia burgdorferi* (Sonenshine et al., 2006). Field trials by Dolan et al. (2011) have revealed that infections rates with *A. phagocytophilum* and *B. burgdorferi* can be significantly reduced in both rodent reservoirs and ticks *I. scapularis* using antibiotic-treated baits. Thus this method can successfully reduce tick infestations and may also help to reduce pathogen transmission but can also contribute to the selection of acaricide and/or antibiotic resistant ticks.

Ecological approaches to control diseases involve intervention in the natural cycle of disease agents vaccinating wild reservoirs but the effects may be complex and hard to predict. For instance, Tsao et al. (2004) immunized white-footed mice, reservoir host for the Lyme disease agent, with a recombinant outer surface protein A (OspA). Even though vaccination significantly reduced the prevalence of *B. burgdorferi* in nymphal ticks, the results also indicated that non-mouse hosts played a larger than expected role in infection dynamics, suggesting the need to vaccinate additional hosts.

Entomopathogenic fungi, such as *Metarhizium anisopliae* and *Beauveria bassiana*, are active against a range of several economically important species of ticks under laboratory and field conditions, for example *R. annulatus* (Pirali-Kheirabadi et al., 2007), *I. scapularis* (Hornbostel et al., 2005), *R. appendiculatus* and *A. variegatum* (Kaaya et al., 1996). Despite the relative safety of this type of biocontrol method they haven't been successfully implemented as yet because of their environmental instability, and potential damage to non-target species.

Ticks can harbor a wide range of endosymbiotic bacteria including *Rickettsia*, *Francisella*, *Coxiella*, and *Arsenophonus*, amongst others (Alberdi et al., 2012). Tick control strategies could be devised based on interference with their endosymbionts for the control of these vectors and the pathogens they hold (Ghosh et al., 2007). For instance, *Wolbachia pipientis* when transfected into *Aedes aegypti* mosquitoes hinders the replication of Dengue and Chikungunya viruses (Iturbe-Ormaetxe et al., 2011).

Vaccination is an attractive alternative for the control of tick infestations and pathogen infections as it is a more environmentally friendly method. By targeting a common vector, several tick-borne diseases can be controlled simultaneously (Brossard, 1998; de la Fuente et al., 1998, 2007a,b, 2011; Rodríguez Valle et al., 2004; Almazán et al., 2005b). Since vector-borne pathogens exploit tick proteins to establish an infection, targeting the pathogen in the vector by blocking transmission is an innovative and promising method to control vector-borne infections (Lee and Opdebeeck, 1999; Havlíková et al., 2009). However, the selection of suitable antigens is a major constraint on vaccine development.

## Finding candidate tick protective antigens

Candidate tick protective antigens have been identified using high throughput screening technologies allowing rapid, systematic and global antigen screening and providing a comprehensive approach for the selection of candidate vaccine antigens (Diatchenko et al., 1999; Almazán et al., 2003; Antunes et al., 2012). Other screening approaches include using RNA interference (RNAi) (de la Fuente et al., 2005, 2008b, 2010; Almazán et al., 2010; Kocan et al., 2011) and capillary feeding (Almazán et al., 2005a; Canales et al., 2009a; Gonsioroski et al., 2012; Rodriguez-Valle et al., 2012). Using a functional genomics approach, Antunes et al. (2012) identified differentially expressed genes in *B. bigemina*-infected *Riphicephalus* ticks. TROSPA and serum amyloid A in particular significantly reduced bacterial infection levels in the ticks. Other methods such as protein arrays (Manzano-Román et al., 2012) and yeast surface display (Schuijt et al., 2011b) have also been proposed for the identification and characterization of antigens that elicit tick immunity.

The tick protective antigen, Subolesin, was discovered by expression library immunization and evaluation of expressed sequence tags (Almazán et al., 2003). Ghosh et al. (2008) employed strategic methods for the isolation of targeted molecules using affinity purification of proteins showing reactivity with immunoglobulins of animals previously immunized with different sources of tick antigens. Rachinsky et al. (2008) investigated the differences in protein expression in midgut tissue of uninfected and *Babesia bovis*-infected *R. microplus* ticks to establish a proteome database containing proteins involved in pathogen transmission. As pathogen neutralization occurs within the feeding vector, the development of a successful transmission-blocking vaccine requires that the antigen induce high and long-lasting circulating antibody titers in immunized hosts.

Nano/microparticle technologies can be applied toward the development of transmission-blocking vaccines that target antigens expressed only inside the vector. Although not yet used in ticks, experiments by Dinglasan et al. (2013) showed that a single inoculation and controlled release of mosquito antigen in mice, elicited long-lasting protective antibody titers against malaria sexual stages. Conserved carbohydrate targets have been identified in the midgut of arthropod species (Dinglasan et al., 2005) and are a promising tool for the elaboration of transmission blocking vaccines that control a wide range of arthropod vectors.

## Specific tick antigens and their effect on pathogen transmission (see table 1)

### BM86-based vaccines

Tick vaccines became commercially available in the early 1990's for the control of cattle tick infestations (Willadsen et al., 1995; Canales et al., 1997; de la Fuente et al., 1998, 2007b). TickGARD (in Australia) and Gavac (in Latin American countries) are both derived from *R. microplus* midgut membrane-bound recombinant protein BM86. The protective action of BM86-based vaccines in cattle is due to the positive correlation between antigen-specific antibodies and reduction of ticks infestations and fertility (Rodríguez et al., 1995; de la Fuente et al., 1998; Merino et al., 2011a). The mechanism by which BM86 immunization affects ticks involves antibody-antigen interaction that interferes with the still unknown BM86 biological function thus reducing the number, weight and reproductive capacity of engorging female ticks (de la Fuente et al., 1998, 1999). As a result, the prevalence of some tick-borne pathogens can indirectly be affected (de la Fuente et al., 2007b). Vaccine trials with BM86 resulted in a reduction in the incidence of babesiosis, as well as reduced tick infestations in vaccinated cattle herds, and these results were corroborated in extensive field trials (de la Fuente et al., 1998, 2007a; Rodríguez Valle et al., 2004). However, because *A. marginale* is also mechanically transmitted by blood-contaminated mouth parts of biting insects and fomites, BM86 antigen vaccination controlled the transmission of *A. marginale* only in regions where ticks are the main vectors (de la Fuente et al., 1998).

**Table 1:** **Overview of tick protective antigens and their effect on the control of tick-borne pathogens**.

**Vaccinated hosts (N)^a^**	**Recombinant tick antigen**	**Vector^b^**	**Pathogen^c^**	**Reduction in vector infection^d^**	**References**
Cattle (>260,000)	Bm86	*R. microplus*	*Babesia sp.*	76%^e^	de la Fuente et al., 1998
Cattle (>260,000)	Bm86	*R. microplus*	*Anaplasma sp.*	No effect	de la Fuente et al., 1998
Cattle (5)	Ba86	*R. annulatus*	*Babesia sp.*	N/D	Canales et al., 2009a
Cattle (5)	Ba86	*R. annulatus*	*Anaplasma sp.*	N/D	Canales et al., 2009a
Rabbit (4)	Bm95	*R. microplus*	*Babesia sp.*	N/D	Canales et al., 2009b
Rabbit (4)	Bm95	*R. microplus*	*Anaplasma sp.*	N/D	Canales et al., 2009b
Cattle (5)	HGAg	*H. a. anatolicum*	*Theileria annulata*	10%	Das et al., 2005; Ghosh et al., 2008;
Cattle (5)	Haa86	*H. a. anatolicum*	*Theileria annulata*	3 calves survived lethal challenge	Jeyabal et al., 2010
Cattle (5)	Bm91	*R. microplus*	*Babesia,*	N/D	Willadsen et al., 1996
Cattle (5)	Bm91	*R. microplus*	*Anaplasma*	N/D	Willadsen et al., 1996
Mice (5)	SUB	*I. scapularis*	*A. phagocytophilum*	33%	de la Fuente et al., 2006b
Cattle (4)	SUB	*R. microplus*	*A. marginale*	98%	Merino et al., 2011b
Cattle (4)	SUB	*R. microplus*	*B. bigemina*	99%	Merino et al., 2011b
Mice (15)	SUB	*I. scapularis*	*B. burgdorferi*	40%	Bensaci et al., 2012
Mice (10)	64TPR	*I. ricinus*	TBEV	52%	Labuda et al., 2006
Rabbits (2); Cattle (4)	RmFER2	*I. ricinus, R. microplus, R. annulatus*	*Anaplasma sp., Babesia sp.*	N/D	Hajdusek et al., 2010
Mice (5)	Salp15	*I. scapularis*	*B. burgdorferi*	60%	Dai et al., 2009
Mice (5)	Salp25D	*I. scapularis*	*B. burgdorferi*	Three-fold	Narasimhan et al., 2007
Cattle (5)	RAS-3, RAS-4, RIM36 coktail	*R. appendiculatus*	*T. parva*	38%	Imamura et al., 2008
Mice (5)	TROSPA	*I. scapularis*	*B. burgdorferi*	75%	Pal et al., 2004
Mice (5)	tHRF	*I. scapularis*	*B. burgdorferi*	20–30% mice fully protected	Dai et al., 2010
Mice (3)	TSLPI	*I. scapularis*	*B. burgdorferi*	30%	Schuijt et al., 2011a

a N, number of individuals per group.

b Arthropod vector species in which vaccine was tested.

c Pathogen species in which the effect of vaccination was tested.

d Reduction in vector infection was determined with respect to the control group vaccinated with adjuvant/saline.

e Overall reduction in the incidence of dead animals caused by infections with Babesia sp. after vaccination.

Abbreviations *N/D* *not determined.*

Despite the effectiveness of these commercial BM86-based vaccines for the control of cattle tick infestations, they show strain-to strain variation in efficacy and are effective against *Rhipicephalus* tick species mainly (de la Fuente and Kocan, 2003; Willadsen, 2006; de la Fuente et al., 2007a,b; Odongo et al., 2007) hence the need to develop improved vaccine formulations (Guerrero et al., 2012).

### BM86 orthologs and homologs

BA86 is a recombinant *R. annulatus* BM86 ortholog protein with over 90% similarity to BM86 (Canales et al., 2008). Experimental trials in cattle proved the efficacy of recombinant BA86 for the control of *R. annulatus* and *R. microplus* infestations, showing that the efficacy of both BM86 and BA86 is higher against *R. annulatus*. These results suggested that physiological differences between *R. microplus* and *R. annulatus* and those encoded in the sequence of BM86 orthologs may be responsible for the differences in susceptibility of tick species to BM86 vaccines (Canales et al., 2009a; Jeyabal et al., 2010).

A BM86 ortholog of *Hyalomma anatolicum anatolicum*, HAA86, was cloned and expressed by Azhahianambi et al. (2009). Jeyabal et al. (2010) reported that vaccination of cattle with the recombinant HAA86 antigen did not only protect against homologous tick challenge but also reduced tick transmission of *Theileria annulata*, thus protecting the animals against lethal exposure.

The *R. microplus* BM95 glycoprotein is a BM86 homologue that protects cattle against infestations by South American cattle tick strains not protected by BM86 vaccination (Canales et al., 2009b). Studies with BM95 have shown it protects against a broader range of tick strain infestations suggesting BM95 could be a more universal antigen against infestations by *R. microplus* strains from different geographical areas (García-García et al., 2000; de la Fuente and Kocan, 2003).

The number of new upcoming promising targets that can affect both tick infestations and pathogen transmission is rising. Nijhof et al. (2010) have recently identified a novel protein from metastriate ticks with structural similarities to BM86, named ATAQ after a part of its signature peptide. Although its function is unknown, ATAQ is expressed in both midguts and Malpighian tubules, while BM86 is expressed only in midguts. The vaccine efficacy of recombinant ATAQ proteins against tick infestations has not been evaluated but it may constitute a good vaccine candidate with an increased cross-protective effect against heterologous ticks compared to BM86-based vaccines because ATAQ proteins are more conserved.

### Tick salivary proteins

Arthropod vectors induce immunosuppression in the host during feeding and secrete pathogen transmission-enhancing factors that counteract host rejection responses. For example, the Lyme disease agent *B. burgdorferi* exploits tick salivary proteins (B-cell inhibitory protein BIP and Salp15 from *I. ricinus* and *I. scapularis*, respectively) to facilitate transmission to the mammalian host (Anguita et al., 2002, 2003; Hannier et al., 2004; Ramamoorthi et al., 2005). During feeding, tick salivary glands secrete a large variety of pharmacologically active molecules with immunosuppresive properties that facilitate pathogen transmission and are potential candidates for anti-tick vaccines that limit infestations and interfere with tick-borne pathogen transmission (Valenzuela, 2002; Ribeiro and Francischetti, 2003; Nuttall et al., 2006; Titus et al., 2006; Nuttall, 2009).

**64TRP** is a 15 kDa protein that resembles mammalian host skin proteins, identified in expression libraries as a putative tick cement protein involved in the attachment and feeding of *R. appendiculatus* (Trimnell et al., 2002; Havlíková et al., 2009). The protein derives from the cement cone that secures the tick's mouthparts in the host skin and, as a broad-spectrum vaccine antigen, is effective against adult and immature stages of several tick species, including *I. ricinus* (Trimnell et al., 2005). Recombinant forms of *R. appendiculatus* 64TRP induce potent humoral and delayed type hypersensitivity responses (Trimnell et al., 2002). In hamster, guinea pig and rabbit models this cement antigen acts as a dual-action vaccine by targeting the tick-feeding site (impairing attachment and feeding) and cross-reacting with “concealed” midgut antigens, resulting in death of engorged ticks (Trimnell et al., 2002, 2005; Havlíková et al., 2009). Histological and immunocytological studies have indicated that the key mode of action of 64TRP immunisation is the local cutaneous delayed type hypersensitivity response induced at the skin site of tick feeding (Labuda et al., 2006). Recent experiments have illustrated how vaccination with this antigen also affects tick vector capacity. Labuda et al. (2006) reported that vaccination of mice with 64TRPP antigen prevented transmission of tick-borne encephalitis virus (TBEV) by *I. ricinus* thus having a protective effect on pathogen transmission.

**Salp15** is another secreted salivary protein with host immunosuppressive properties, inhibiting CD4^+^ T-cell activation (Anguita et al., 2002), complement activity (Schuijt et al., 2008), and dendritic cell function (Hovius et al., 2008a). OspC is an outer surface protein produced by *B. burgdorferi*. When ticks take a blood meal, the spirochetes initiate its synthesis in the midguts of infected ticks. Salp15 physically binds to OspC on *B. burgdorferi* spirochetes surface during exit from the salivary glands, facilitating the survival of spirochetes, pathogen transmission and host infection (Ramamoorthi et al., 2005; Dai et al., 2009). Salp15-OspC interaction potentially conceals OspC from the host immune response protecting the spirochete (Ramamoorthi et al., 2005). Mice immunized with recombinant Salp15 and challenged with *B. burgdorferi* infected nymphs were significantly protected from infection (Dai et al., 2009). Antibodies directed against Salp15 may separate Salp15 away from OspC leaving it exposed to the immune responses, or, hypothetically, Salp15 antibodies could bind to Salp15-coated spirochetes and release the spirochetes more effectively to phagocytes (Dai et al., 2009). Immunization of murine hosts with a combination of Salp15 and OspA provide better protection from *B. burgdorferi* infection than either alone (Dai et al., 2009). Salp15 homologs have been identified in *I. ricinus* ticks, they also bind *B. garinii* and *B. afzelii* OspC to facilitate spirochete transmission (Hovius et al., 2008b).

**Salp25D** is expressed *by I. scapularis* salivary glands and midguts (Das et al., 2001) and has homology to peroxiredoxins antioxidants (Barr and Gedamu, 2003). Immunization of mice with rSalp25D reduces *Borrelia* acquisition by *I. scapularis* (Narasimhan et al., 2007) demonstrating it plays a critical role during tick feeding in the mammalian host, protecting the bacteria from reactive oxygen produced by neutrophils and facilitating *Borrelia* acquisition by ticks. Therefore it could be used to vaccinate reservoir hosts to interrupt the spirochete life cycle and reduce its prevalence in ticks in Lyme disease endemic areas. Interestingly, Salp25D does not influence transmission from the tick to the mammalian host (Narasimhan et al., 2007).

The **tick histamine release factor (tHRF)** from *I. scapularis* was characterized by Dai et al. (2010). tHRF is secreted in tick saliva, upregulated in *B. burgdorferi*-infected ticks and it appears to have a role in tick engorgement and efficient *B. burgdorferi* transmission (Dai et al., 2010). Silencing tHRF by RNAi significantly impaired tick feeding and decreased *B. burgdorferi* infection levels in mice. Actively immunized mice with recombinant tHRF, or passively transferring tHRF antiserum, also markedly reduced the efficiency of tick feeding and *B. burgdorferi* infection in mice. Blocking tHRF might offer a viable strategy to develop vaccines that block tick feeding and therefore transmission of tick-borne pathogens.

The *I. scapularis* salivary protein **TSLPI (Tick Salivary Lectin Pathway Inhibitor)** identified by Schuijt et al. (2011a) protects *B. burgdorferi* from direct killing by the host complement system. Silencing *TSLPI* mRNA significantly reduces *Borrelia* loads in nymphs and also impairs transmission to mice. TSLPI plays a significant role in both transmission and acquisition of *Borrelia* (Schuijt et al., 2011a) but immunization against rTSLPI does not completely block bacterial transmission from the tick to the host, suggesting the need for a combination of tick proteins in future tick antigen-based vaccines to prevent Lyme disease (Schuijt et al., 2011b).

### Other tick proteins

**Ferritins** are iron-storage proteins that play a pivotal role in the homeostasis of iron during tick feeding. A common heavy chain type ferritin 2 (Kopacek et al., 2003), without functional orthologs in vertebrates, has been recently characterized as a gut-specific protein secreted into the tick hemolymph, where it acts as an iron transporter (Hajdusek et al., 2009). Ferritin 2 (RmFER2) knockdown by RNAi and vaccination with the recombinant protein resulted in reduction of feeding, oviposition and fertility in *I. ricinus, R. microplus* and *R. annulatus* (Hajdusek et al., 2009, 2010) thus highlighting its potential use as a future dual action tick and tick-borne diseases protective antigen candidate.

**TROSPA** is a tick receptor for *B. burgdorferi* OspA that has been identified in the tick midgut (Pal et al., 2004; Antunes et al., 2012). Tick-borne pathogens can adapt from the vector to the mammalian host by differential gene expression. For example, outer surface proteins OspA and OspB are produced when Lyme disease spirochetes enter and reside in ticks (Pal and Fikrig, 2003) but they are downregulated during transmission to the host. Other genes that facilitate transmission from ticks and colonization of the host such as bba52 and OspC are upregulated. TROSPA expression is upregulated during *B. burgdorferi* infection and downregulated during tick engorgement. The receptor's physiological function is unknown but binding of OspA to TROSPA is essential for *B. burgdorferi* to colonize the tick gut, thus supporting bacterial infection in the vector (Pal et al., 2004). *B. burgdorferi* infection enhances expression of specific tick genes such as TROSPA and salp15 that can be targeted to prevent the transmission of *Borrelia* spirochetes and other tick-borne microbes (Hovius et al., 2007). Blocking TROSPA with TROSPA antisera or via RNA interference (RNAi) reduces *B. burgdorferi* adherence to the gut of *I. scapularis*, and as a result reduces bacterial colonization of the vector and, potentially, pathogen transmission to the host (Pal et al., 2004). Bacterial OspA has been used as a Lyme disease vaccine that blocks pathogen transmission as anti-OspA antibodies destroy the spirochetes in the tick gut before transmission to the host occurs (Pal et al., 2000). Studies by Tsao et al. (2001) suggested that vaccination of mice with OspA could reduce transmission of the bacteria to the tick vector regardless of whether the reservoir host was previously infected or not. A combination of OspA with TROSPA antigens may enhance vaccine protective efficacy against Lyme disease.

**Serpins** (serine protease inhibitors) are a large family of structurally related proteins found in a wide variety of organisms, including hematophagous arthropods. They are known to regulate many important functions such as blood coagulation, food digestion, inflammatory and immune responses (Mulenga et al., 2001) and therefore are attractive target antigens for tick vaccine development. Combining different serpins to vaccinate cattle results in a reduction of engorgement rates and increased mortality of *Haemaphysalis* and *Rhipicephalus* ticks (Imamura et al., 2005, 2006). Furthermore, immunization of cattle with a cocktail vaccine containing recombinant *R. appendiculatus* serpins RAS-3, RAS-4, and a 36 kDa immune-dominant protein RIM36, reduces tick infestations and also has an effect on the tick mortality rate of *Theileria parva*-infected ticks by increasing it from 10.8 to 48.5% in the vaccinated group (Imamura et al., 2008). Infection of cattle with *T. parva* could not be prevented by the vaccine although the presence of the pathogen in peripheral blood was delayed by a couple of days indicating the vaccine also had an effect on pathogen transmission to the mammalian host.

**Figure 2:**
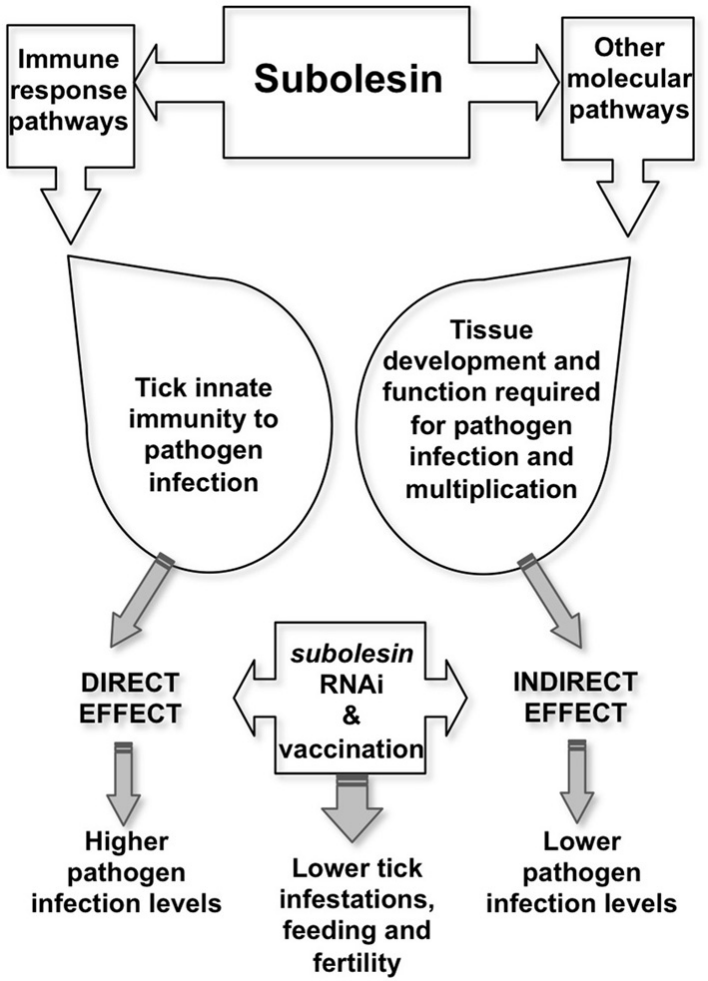
**Model for Subolesin role in pathogen infection**. Targeting SUB by vaccination or RNAi reduces tick immunity, thereby increasing pathogen infection levels. However, lower pathogen infection levels may result from the effect on tissue structure and function and the expression of genes that are important for pathogen infection and multiplication. Both direct and indirect effects of targeting SUB results in lower tick infestations, feeding and fertility.

**Tick Subolesin (SUB)**, the ortholog of insect and vertebrate akirins (AKR) (de la Fuente et al., 2006a; Goto et al., 2008; Canales et al., 2009c; Galindo et al., 2009; Macqueen and Johnston, 2009; Mangold et al., 2009), was discovered as a tick protective antigen in *I. scapularis* (Almazán et al., 2003). Most vertebrates have two closely related AKR homologues, AKR1 and AKR2 (Goto et al., 2008). Only one SUB/AKR gene has been identified in insects and ticks, which is evolutionary and functionally related to mammalian AKR2 (de la Fuente et al., 2006a; Goto et al., 2008; Galindo et al., 2009; Macqueen and Johnston, 2009). SUB has a role in tick immunity and other molecular pathways and has been shown to protect against tick infestations and infection by vector-borne pathogen such as *A. phagocytophilum*, *A. marginale*, *B. bigemina*, and *B. burgdorferi* (de la Fuente et al., 2006b; Merino et al., 2011b; Bensaci et al., 2012). RNAi experiments have demonstrated that SUB knockdown affects the expression of genes involved in multiple cellular pathways (de la Fuente et al., 2006c, 2008c). It also has an effect on pathogen infection by reducing tick innate immunity that results in higher infection levels but also indirectly by affecting tick tissue structure and function and the expression of genes required for pathogen infection, therefore interfering with pathogen infection and multiplication (Zivkovic et al., 2010; de la Fuente et al., 2011) (Figure 2). Vaccines containing conserved SUB/AKR protective epitopes have been shown to protect against tick, mosquito and sand fly infestations, thus suggesting the possibility of developing universal vaccines for the control of arthropod vector infestations (Moreno-Cid et al., 2013). However, the effects of SUB/AKR vaccines on vector-borne viruses showed no effect on tick-borne encephalitis virus infection and transmission (Havlíková et al., 2013).

## Conclusions and future directions

This review has focused on studies showing the effects of tick antigens on the control of tick-borne pathogens by either decreasing the exposure of susceptible hosts to infected ticks (i.e., BM86) or by reducing tick vector capacity (i.e., TROSPA) (Kocan, 1995; de la Fuente and Kocan, 2003; Willadsen, 2006; de la Fuente et al., 2007a,b).

Tick-borne pathogens are maintained in a complex enzootic infection cycle involving ticks and vertebrate hosts (Wilson, 2002). Our understanding of the biology of vector-pathogen interactions, primarily involving model insects has advanced over the past decades. However, our knowledge of tick biology, especially the molecular interactions with the pathogens they maintain and transmit, and the mechanism by which the tick immune response influences invading pathogens, remains insufficient. The relative fitness of a pathogen within the vector can be a major determinant of pathogen prevalence within the vertebrate host population. For example, strains of the tick-borne rickettsia *A. marginale* differ markedly in their transmission efficiency (Ueti et al., 2009). These areas are understudied but important and warrant future investigation.

Transmission-blocking vaccines that interfere with specific aspects of tick physiology important for arthropod survival or development may prevent multiple infections that are often co-transmitted by a single tick species, an advantage over vaccines which only target particular pathogens. For example, immunization of hosts using SUB significantly inhibits tick infection with multiple pathogens such as *A. marginale* and *B. bigemina* (Merino et al., 2011b).

Progress in the development of transmission blocking vaccines has been slow. The limiting step in the development of vector vaccines has been the identification of new antigens that induce protective immune responses whilst preventing pathogen transmission (de la Fuente and Kocan, 2003). The number of proteins that may be of value as antigens has continued to increase quite rapidly over recent years but there have not been many reports of their actual assessment in vaccination trials (Willadsen, 2004; Guerrero et al., 2012). Very few antigens appear to be highly effective on their own suggesting the need for a multi-antigen or chimeric vaccine that incorporates critical tick and pathogen antigenic epitopes (Almazán et al., 2012; Parizi et al., 2012b; Moreno-Cid et al., 2013) to elicit synergistic anti-pathogen and anti-tick immune responses.

The selection of new vaccine antigens from the study of tick-pathogen interactions using systems biology requires the development of algorithm that allow the selection of the most effective targets to control tick infestations and pathogen transmission (de la Fuente, 2012).

Finally, identification of new protective antigens that are conserved across vector species, with similar structure and/or sequence motifs, may provide the opportunity to develop a universal and so more commercially viable vaccine for the control of multiple arthropod infestations and their associated pathogens (de la Fuente et al., 2011; Parizi et al., 2012a; Moreno-Cid et al., 2013).

### Conflict of interest statement

The authors declare that the research was conducted in the absence of any commercial or financial relationships that could be construed as a potential conflict of interest.
